# Correlation Between Structural Distortion and Dielectric–Magnetic Properties in Ba-Doped Multiferroic BiFeO_3_

**DOI:** 10.3390/ma19183861

**Published:** 2026-09-10

**Authors:** Emna M. Benali, Manuel P. F. Graça, Essebti Dhahri, Benilde F. O. Costa, Adel Benali

**Affiliations:** 1 Laboratory of Applied Physics, Faculty of Sciences of Sfax, University of Sfax, B.P. 1171, Sfax 3000, Tunisia; benaliemna93@gmail.com (E.M.B.); essebti@yahoo.com (E.D.); 2 University of Coimbra, CFisUC, Physics Department, 3004-516 Coimbra, Portugal; 3 i3N, Physics Department, University of Aveiro, Campus de Santiago, 3810-193 Aveiro, Portugal; mpfg@ua.pt

**Keywords:** multiferroic, nanoparticles, dielectric relaxation, colossal dielectric constant, magnetization

## Abstract

Bi_0.8_Ba_0.2_FeO_3_ nanoparticles were synthesized using the sol–gel method. X-ray diffraction (XRD) analysis confirmed that the material crystallizes in a tetragonal structure with the P4/mmm space group. This structural transformation plays an important role in the microstructural distortion and phase modification of the material, which are favorable for enhancing the magnetic and dielectric properties. The substitution of Ba^2+^ ions in the Bi-site of the BiFeO_3_ lattice leads to a notable increase in the remanent (Mᵣ) and saturation magnetization (M_S_) along with the coercive field (H_C_). Furthermore, the Bi_0.8_Ba_0.2_FeO_3_ compound exhibits a high dielectric constant ($\varepsilon^{\prime }$) accompanied by a low dielectric loss (tg(δ)), manifesting its strong potential for applications in capacitors and dynamic random-access memory (DRAM) devices.

## 1. Introduction

Multiferroic materials, distinguished by the coexistence of two or more ferroic order parameters, mainly ferroelectricity, ferromagnetism, and ferro-elasticity, have attracted significant research interest given their potential applications in multifunctional devices including sensors, spintronic devices, data storage, and actuators [1,2]. Among these materials, bismuth ferrite (BiFeO_3_) has emerged as one of the most extensively studied single-phase multiferroics as it features both ferroelectric and antiferromagnetic ordering at room temperature [3].

BiFeO_3_ crystallizes in a distorted perovskite structure with rhombohedral symmetry (space group *R3c*). It possesses a high ferroelectric Curie temperature (~1103 K) and an antiferromagnetic Néel temperature (~643 K), making it particularly attractive for room-temperature applications [3,4]. However, bulk BiFeO_3_ shows a cycloidal spin structure with a long wavelength (~62 nm), which cancels out macroscopic magnetization and limits the material’s practical magnetic functionality [5].

Reducing BiFeO_3_ to the nanoscale has been shown to significantly affect its dielectric and magnetic properties. In BiFeO_3_ nanoparticles, the suppression of the spiral spin structure, increased surface-to-volume ratio, and enhanced lattice strain can lead to improved magnetization and altered dielectric behavior [6,7]. Additionally, nanoscale effects such as grain boundary polarization, space-charge effects, and defect states, particularly oxygen vacancies, play a crucial role in determining the dielectric response [8].

The dielectric properties of BiFeO_3_ nanoparticles, including dielectric constant, dielectric loss, and frequency dependence, provide valuable insights into polarization mechanisms, charge transport, and relaxation processes [9]. These characteristics are highly sensitive to particle size, synthesis method, and microstructural features, which directly influence their applicability in capacitors and energy-storage devices [10].

Similarly, the magnetic properties of BiFeO_3_ nanoparticles are strongly affected by size reduction, surface effects, and structural distortions. Enhanced weak ferromagnetism, commonly observed in nanoparticulate BiFeO_3_, is attributed to uncompensated surface spins and the breakdown of long-range antiferromagnetic ordering [11,12].

Moreover, the partial ionic substitution at the A-site (Bi^3+^) and/or B-site (Fe^3+^) of BiFeO_3_ was proven to be an effective approach to improving its dielectric and magnetic properties. This can be achieved by modifying the structural distortion, defect chemistry, and magnetic exchange interactions. A-site substitution with rare-earth ions, specifically La^3+^, Gd^3+^, or Pr^3+^, reduces lattice strain arising from the stereo-chemically active Bi^3+^ lone pair. This procedure suppresses secondary impurity phases, and considerably improves the dielectric constant while lowering the dielectric loss through reduced oxygen vacancy concentration and leakage current density [3,13,14]. Correspondingly, B-site substitution with transition metal ions (e.g., Mn, Co, Ni, or Ti) alters the Fe–O–Fe super exchange interactions and local electronic environment, resulting in an enhanced magnetic hysteresis behavior and modified dielectric relaxation mechanisms [15,16].

Recently, it was established that Barium (Ba^2+^) substitution at the A-site of BiFeO_3_ has been widely investigated as an effective mean to amplify both dielectric and magnetic properties by inducing structural distortion and modifying defect chemistry. The incorporation of the larger Ba^2+^ ion in the place of Bi^3+^ leads to lattice expansion and a partial structural transition from the rhombohedral *R3c* phase toward a pseudo-cubic or orthorhombic symmetry, which suppresses secondary impurity phases and stabilizes the perovskite structure [17,18,19]. From a dielectric perspective, Ba substitution significantly increases the dielectric constant and reduces dielectric loss, primarily related to decreased leakage current, reduced oxygen vacancy concentration, and enhanced Maxwell–Wagner interfacial polarization at grain boundaries [20,21]. Furthermore, V. Srinivas et al. reported that the enhancement in the magnetization of the Bi_1-x_Ba_x_FeO_3_ (0 ≤ x ≤ 0.3) nanopowders may be due to the suppression of spiral spin structure caused by the Ba^2+^ ions [22].

On the other hand, compared with other divalent ions (Ca^2+^, Pb^2+^ and Sr^2+^), the substitution with Ba^2+^ ions presents a distinctive advantage due to their larger ionic radius. A. Khomchenko et al. confirmed that the dopant with the biggest ionic radius (Ba^2+^ ions) modifies the spiral structure (structural distortion) of the BiFeO_3_ sample and enhances its magnetic properties, dielectric response, and ferroelectric polarization at room temperature [23].

In this framework, Ba-substituted BiFeO_3_ exhibits a strong correlation between structural modification, improved dielectric response, and enhanced magnetic behavior, making it a promising candidate for multifunctional multiferroic applications.

In this context, the present study focuses on a systematic investigation of the dielectric and magnetic properties of Ba-doped BiFeO_3_ nanoparticles, aiming to establish correlations between nanoscale structure and multifunctional behavior for advanced multiferroic applications.

## 2. Experimental Details

### 2.1. Sample Preparation

Bi_0.8_Ba_0.2_FeO_3_ samples were synthesized by the sol–gel route described in our previous study [23]. Following the typical procedure, stoichiometric amounts of Bismuth nitrate Bi(NO_3_)_3_.5H_2_O, Iron nitrate Fe(NO_3_)_3_.9H_2_O and Barium nitrate Ba(NO_3_)_2_ were dissolved in distilled water. The obtained solution was kept under magnetic stirring until the formation of a homogeneous viscous solution which confirmed, the complete dissolution of all starting materials. Then, citric acid was added to the solution as a chelation agent with a metal: citric acid ratio equal to 1:2.

We added a stoichiometric amount of ethylene glycol (C_2_H_6_O_2_) to the solution and stirred at 70 °C until the formation of a gel. Then, the gel was heated at 180 °C in order to facilitate its transformation into black powder.

Finally, the obtained powder was ground using an agate mortar and calcined at temperatures of 300 °C and 800 °C for 12 h and 30 min, respectively.

### 2.2. Characterization Tools

The X-ray diffraction (XRD) measurements of our prepared sample were carried out using a Siemens D5000 diffractometer (Dresden, Germany) with Cu–Kα radiation (λ = 0.154184 nm) in the range of 2θ = 10–90° at room temperature.

FEI Tecnai G2 transmission electron microscopy (TEM) (Thermo Fisher Scientific, Waltham, MA, USA) was used utilized to analyze the particle size distribution and surface structure.

For the dielectric measurements of our ceramic, two conductive silver layers were coated on both sides of the pellets (using our prepared powder). Then, the transport properties of the studied materials were measured using an Agilent 4294A in the frequency range 100 Hz–1 MHz and the temperature range 120 K–350 K. Finally, the magnetic properties were measured using a vibrating sample magnetometer (VSM) PPMS DynaCool, Quantum Design, Germany, at room temperature.

## 3. Results and Discussions

### 3.1. Structural and Morphological Properties

The room-temperature X-ray diffraction (XRD) measurements were carried out to investigate the structural characteristics of the doped BiFeO_3_ ceramics. Let us recall that the pure BiFeO_3_ sample exhibits exhibit a rhombohedral perovskite structure (space group *R3c*) [22,24]. We can note that the crystal structure of BiFeO_3_ is highly influenced by the A-site and B-site substitution and may transform from a rhombohedral (*R3c*) phase to a tetragonal (*P4mm*) depending on the type of dopant introduced [25].

**Figure 1:**
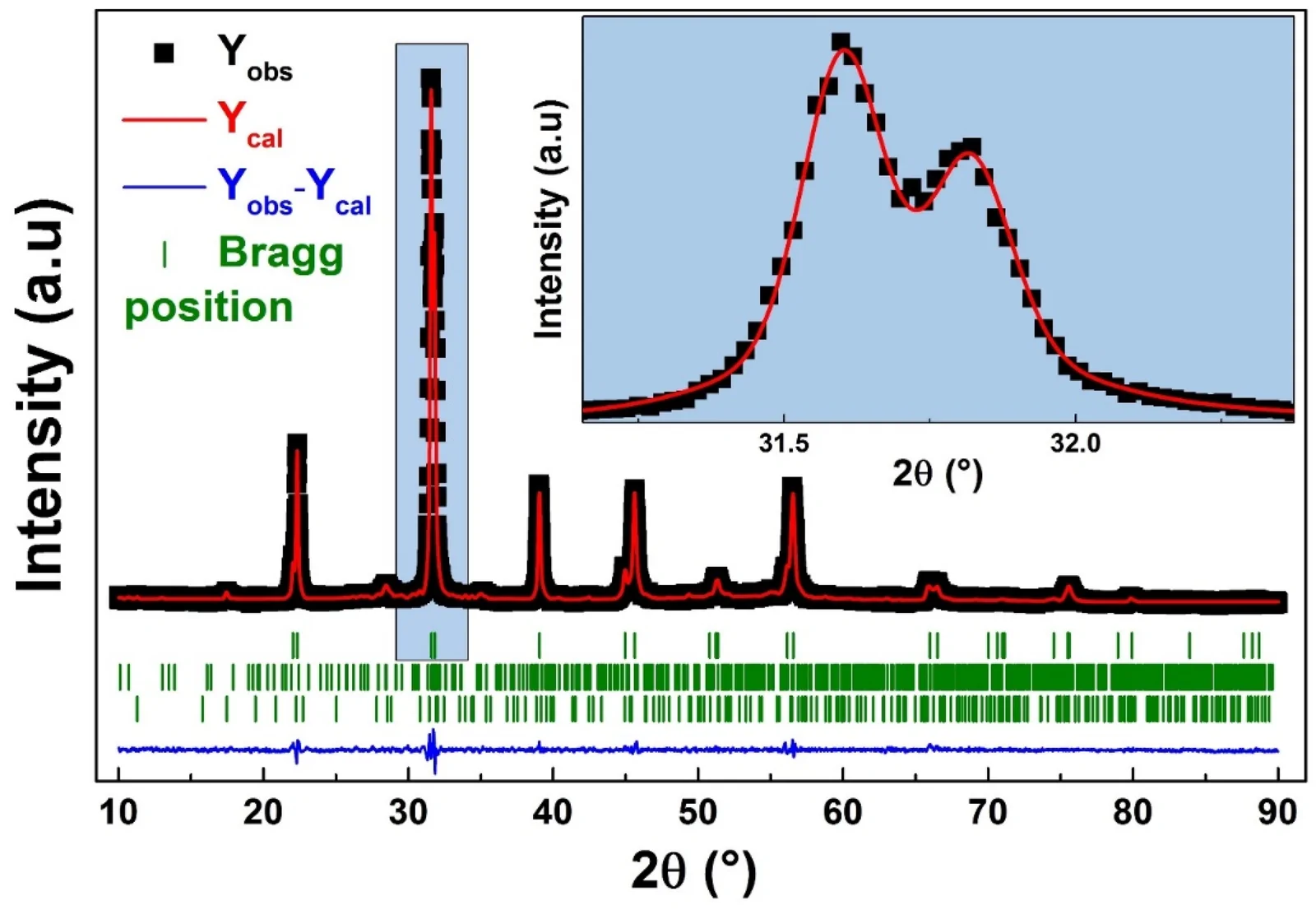
Rietveld Profile refinement of the Bi_0.8_Ba_0.2_FeO_3_ compound.

In this context, Figure 1 shows the XRD pattern of the Ba-doped BiFeO_3_ sample. It is important to mention that the (104) and (110) diffraction peaks (near 32°) merge into one single peak. This behavior is associated with the substitution of Bi^3+^ ions with a smaller ionic radius (1.17 Å) for larger Ba^2+^ ions (1.42 Å) in the BiFeO_3_ lattice [25], which immediately highlights a possible structural transition for the prepared sample.

In our case, the main peaks of the Bi_0.8_Ba_0.2_FeO_3_ ceramic are indexed to the tetragonal structure with the *P4/mmm* space group, which is consistent with our previous study [26]. Nevertheless, the Ba-doped BiFeO_3_ sample contained traces of the secondary phase BaFe_2_O_4_ indexed to the orthorhombic structure and Pbam space group. A further secondary phase was also detected (peak at around 17.8°) and was assigned to the Fe_2_O_3_ phase (*Pbma*).

The X-ray diffraction data of the Bi_0.8_Ba_0.2_FeO_3_ sample were analyzed by Rietveld refinement using the FullProf software, 2021 (Figure 1). The obtained refined parameters are collected in Table 1.

**Figure 2:**
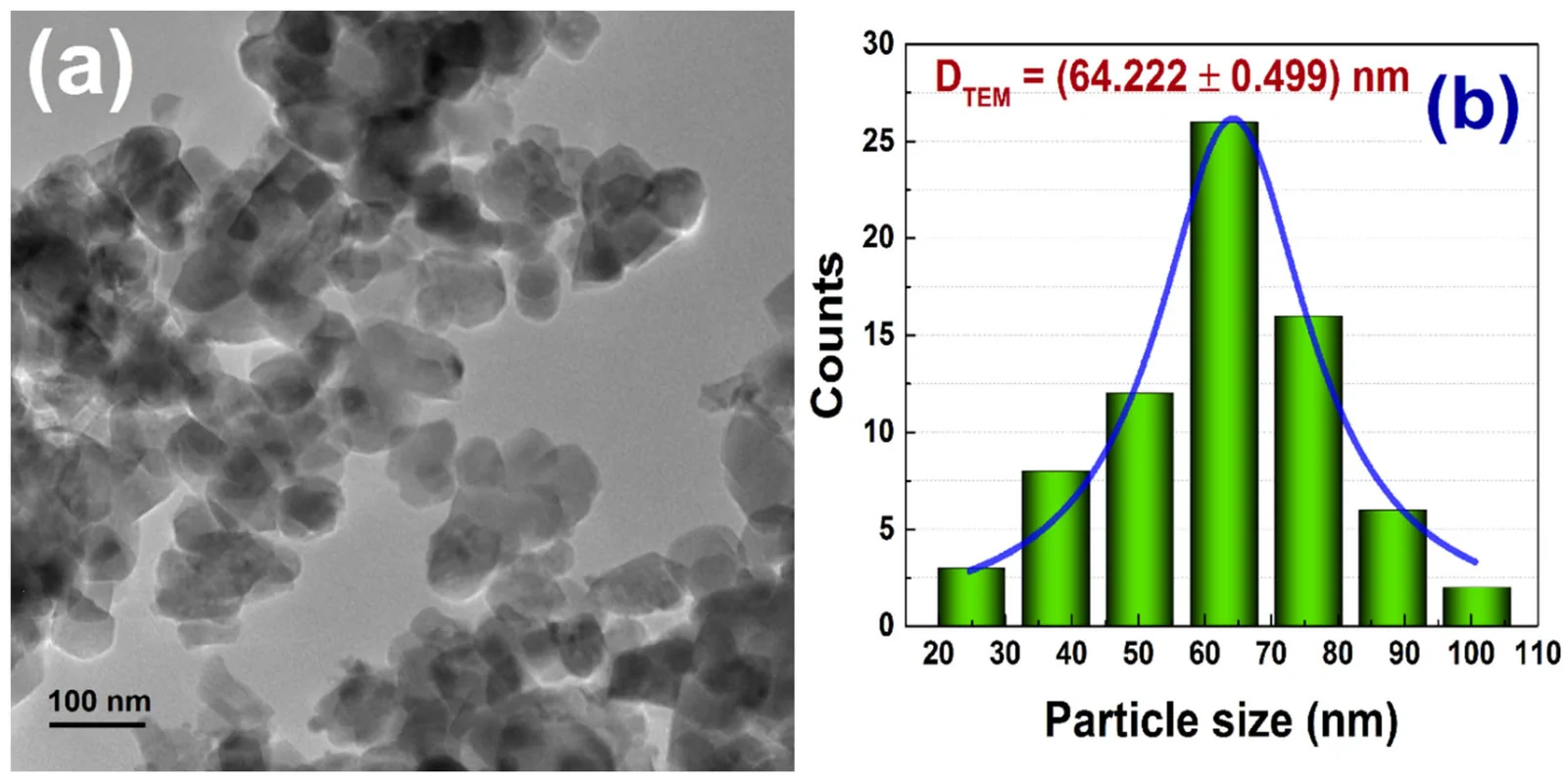
(**a**) TEM morphology image. (**b**) Lorentzian fit of the particle size distribution of the Bi_0.8_Ba_0.2_FeO_3_ compound.

Complementing the structural insights gained from the diffraction data, morphological analysis was deduced from the TEM image of the studied compound. Figure 2a reveals a nanoscale morphology of the nanoparticles, which mostly exhibit irregular polyhedral and quasi-spherical forms, along with some agglomeration. Such aggregation is frequently seen in nanostructured metal oxides and may result from interparticle interactions as well as the elevated surface energy connected to the smaller particle size. With a mean particle size of approximately 64.22 nm, the particle-size distribution shown in Figure 2b has a relatively broad profile and is well-described by a Gaussian function. These findings offer more proof that nanosized Bi_0.8_Ba_0.2_FeO_3_ particles have formed.

### 3.2. Magnetic Properties

It should be emphasized that bulk BiFeO_3_ is widely recognized as an antiferromagnetic material at room temperature, as evidenced by the nearly linear magnetization–field (M–H) curves typically observed for this compound [27,28]. This response is attributed to its G-type antiferromagnetic ordering combined with a long-period cycloidal spin structure, which results in the cancelation of any net macroscopic magnetization [28]. As a consequence, undoped BiFeO_3_ generally show a very low magnetic moment under an applied magnetic field. For example, a room-temperature magnetization of about 0.34 emu/g was reported by S.V. Vijayasundaram et al. [29]. Any departure from this characteristic linear behavior, particularly the emergence of enhanced magnetization, is therefore evidence of modifications in the magnetic structure, which may arise from lattice distortion, defect formation, or dopant-induced suppression of the spin cycloid.

**Figure 3:**
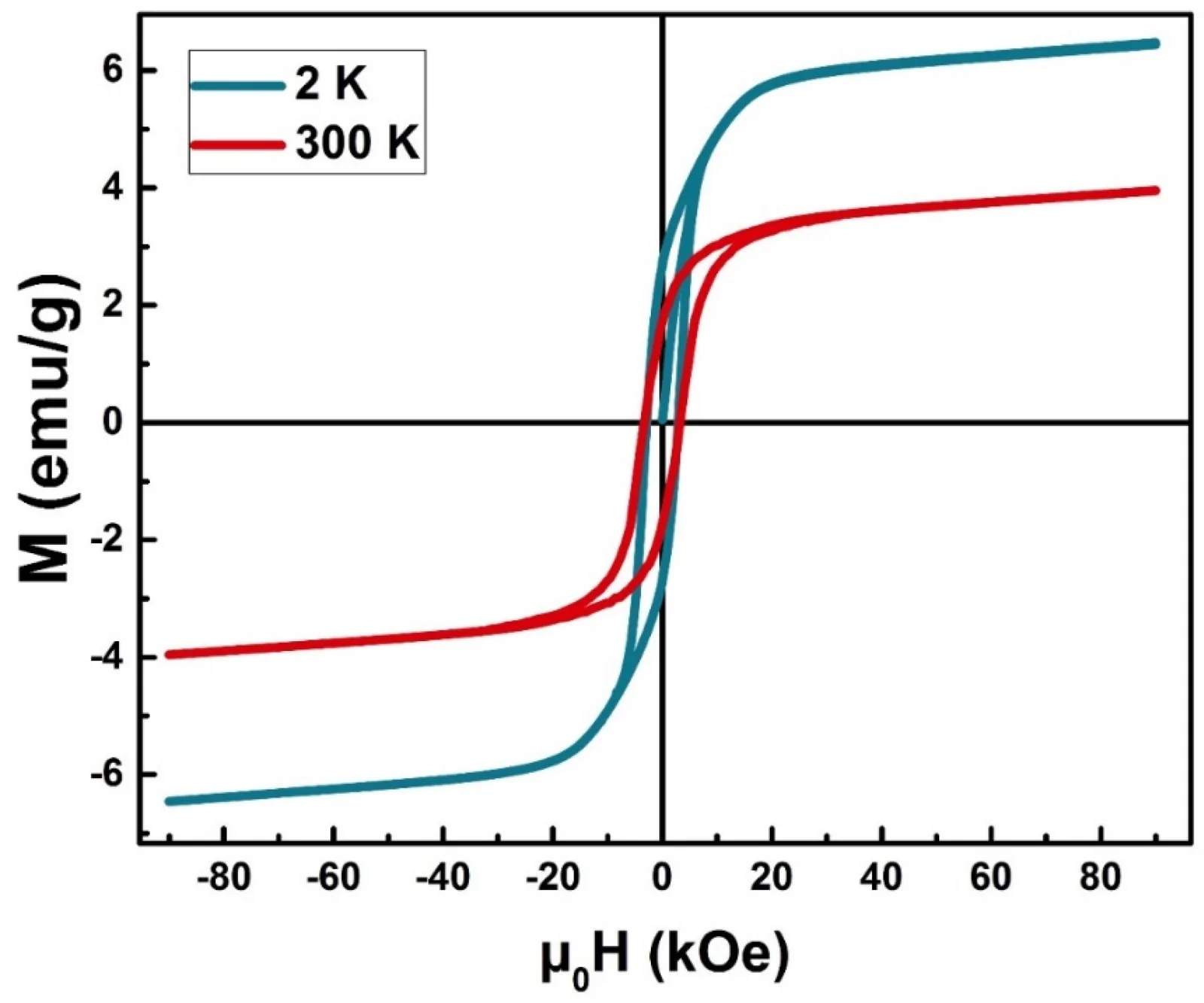
Magnetic hysteresis loops of the Bi_0.8_Ba_0.2_FeO_3_ compound recorded at 2 K and at room temperature.

Figure 3 presents the magnetic hysteresis loops measured at 2 K and at room temperature under an applied magnetic field (μ_0_H) ranging from −9 to +9 T. The magnetic response of the studied compound reveals a pronounced hysteresis loop with measurable remanent magnetization (M_R_) and coercivity (H_C_), revealing a magnetic response dominated by a ferromagnetic component in the Bi_0.8_Fe_0.2_FeO_3_ compound, with a saturation magnetization of approximately 4 emu/g (almost 12 times higher than that of the bulk material [29]). This value is significantly higher than those reported for other doped BiFeO_3_ systems [30,31,32,33,34]. The observed enhancement in magnetization is primarily governed by two key mechanisms. The first is the structural transformation from the rhombohedral *R3c* symmetry, characteristic of pristine BiFeO_3_, to a tetragonal structure belonging to the P4/mmm space group. This structural evolution leads to pronounced lattice distortions, resulting in modifications of the Fe–O–Fe bond lengths and bond angles, which directly affect the super exchange interactions between neighboring Fe^3+^ ions and favor the development of spin canting [35]. The second contributing factor is the partial substitution of Bi^3+^ ions by Ba^2+^ ions at a concentration of 20%. Previous studies have shown that the incorporation of Ba^2+^ can partially reduce Fe^3+^ to Fe^2+^, creating additional Fe^3+^–O–Fe^2+^ super exchange interactions [36,37]. These new magnetic interactions contribute to the strengthening of the ferromagnetic component and lead to an overall enhancement of the material’s magnetization.

Such effects disrupt the long-range antiferromagnetic ordering and suppress the cycloidal spin modulation, thereby reinforcing the ferromagnetic component. The combined influence of structural phase transition and Ba^2+^ incorporation therefore plays an essential role in promoting the dominant ferromagnetic behavior observed in the studied material.

**Figure 4:**
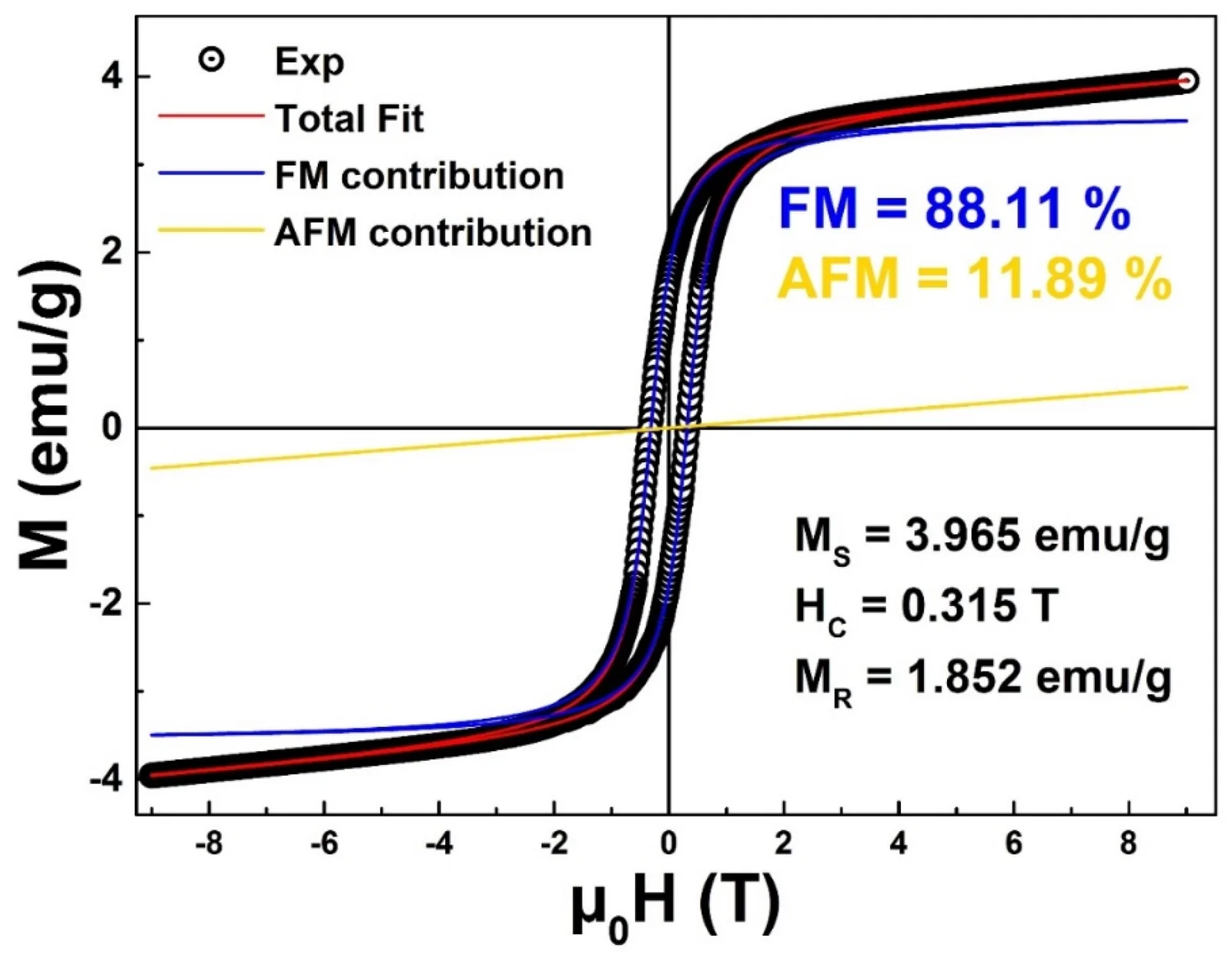
Fit of the room temperature magnetic hysteresis loop of the Bi_0.8_Ba_0.2_FeO_3_ compound.

To boost our assumption in the dominance of the ferromagnetic interactions over the antiferromagnetic ones in the Bi_0.8_Ba_0.2_FeO_3_ nanoparticles, we performed an adjustment of the magnetic hysteresis loop at room temperature according to the following equation [38]:

(1) $$M\left(H\right)=\left\{2\frac{M_{FM}^{S}}{\pi }\tan^{-1}\left(\left(\frac{H\pm H_{ci}}{H_{ci}}\right)\tan\left(\frac{\pi \times M_{FM}^{R}}{2\times M_{FM}^{S}}\right)\right)\right\}+\left\{\chi H\right\}$$

where $H_{CI}$ represents the coercive field, $M_{FM}^{R}$ means the remanent magnetization, and the saturation magnetization is denoted by $M_{FM}^{S}$. The fitting yields two distinct curves, each representing either the ferromagnetic (blue line curve) or antiferromagnetic contribution (yellow line curve), as illustrated in Figure 4. The resulting curve, obtained as the sum of both contributions, is shown as a red line, which closely aligns with the experimental data depicted in dark color in Figure 4.

About 88.11% of the total magnetism in the Bi_0.8_Ba_0.2_FeO_3_ compound under study originates from the ferromagnetic contribution, whereas only 11.89% comes from the antiferromagnetic component. These findings indicate that the magnetic behavior of the compound is largely governed by ferromagnetic interactions, resulting from the partial substitution of the Bi^3+^ ions by Ba^2+^ ions, which explains the magnetic enhancement.

### 3.3. Dielectric Study

#### 3.3.1. Dielectric Constant and Dielectric Loss Tangent

**Figure 5:**
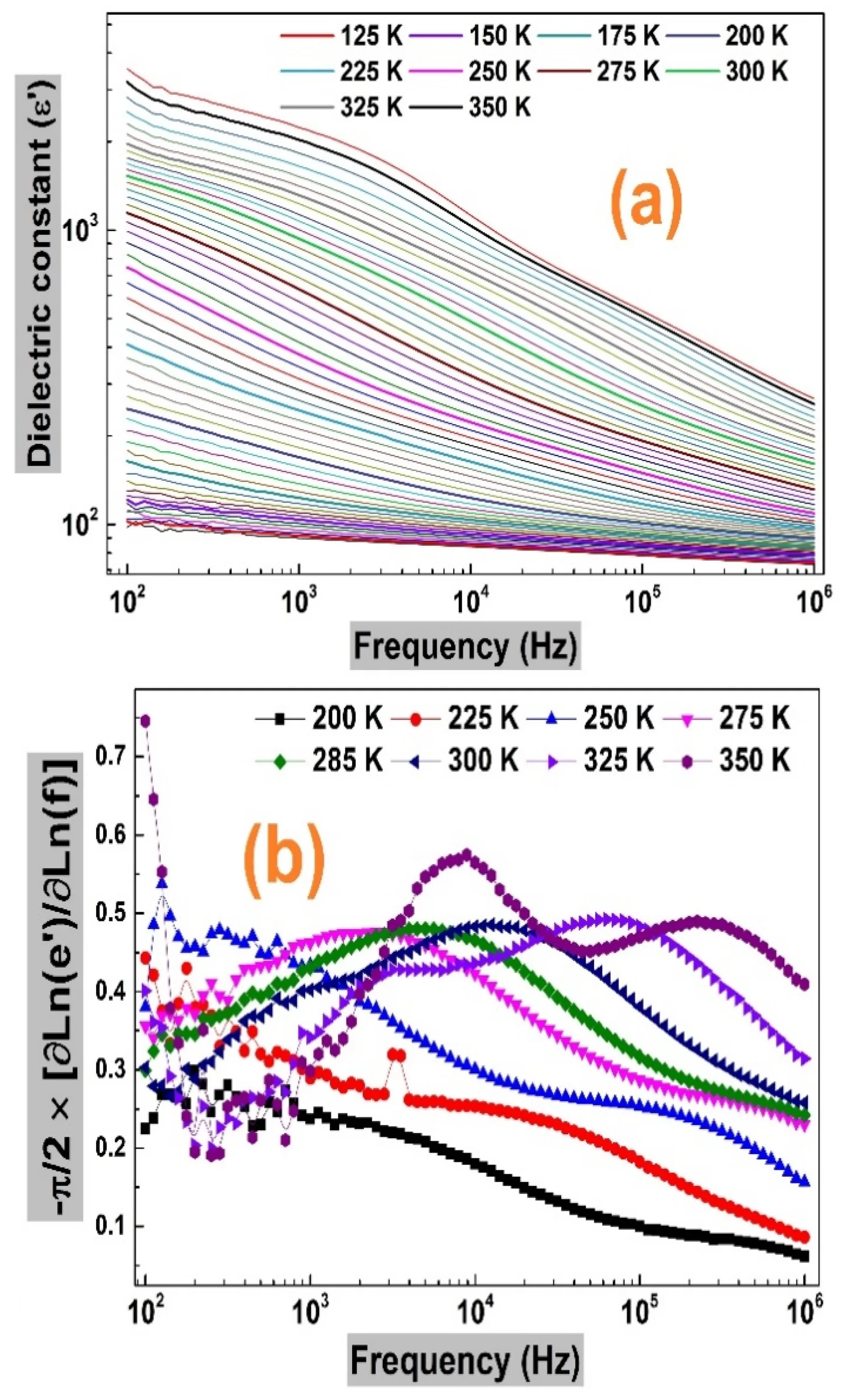
(**a**) Dielectric constant ε’ vs. frequency at different temperatures of the Bi_0.8_Ba_0.2_FeO_3_ compound. (**b**) the corresponding Kramers–Kronig-transformed spectra of the dielectric constant.

The real part of the complex dielectric permittivity ε′, known as the dielectric constant, was calculated and plotted in Figure 5a) over a frequency range of 100 Hz to 1 MHz at various temperatures. As we can clearly see, the dielectric constant exhibits high values at low frequencies (≥10^3^) and decreases progressively with increasing frequency.

This behavior is consistent with previous reports of bulk and doped BiFeO_3_ compounds [26,39,40,41,42,43], which reveal a giant dielectric constant. Notably, three approximate plateaus appear in the dielectric response at around ε’ ≈ 94, 570 and 2.10^3^, while the dielectric constant remains independent of frequency. Between these plateaus, the dielectric constant shows stepwise decreases from one plateau to another. At each temperature, the inflection points between two plateaus indicate the occurrence of a relaxation phenomenon [44]. To further confirm the presence of the relaxation process in the studied compound, we plotted in Figure 5b the logarithmic derivative of the permittivity part (known as the Kramers–Kronig transformation [45,46,47]).

The Kramers–Kronig-transformed spectra confirm the presence of three distinct relaxation peaks, which are attributed to two different contributions [43].

**Figure 6:**
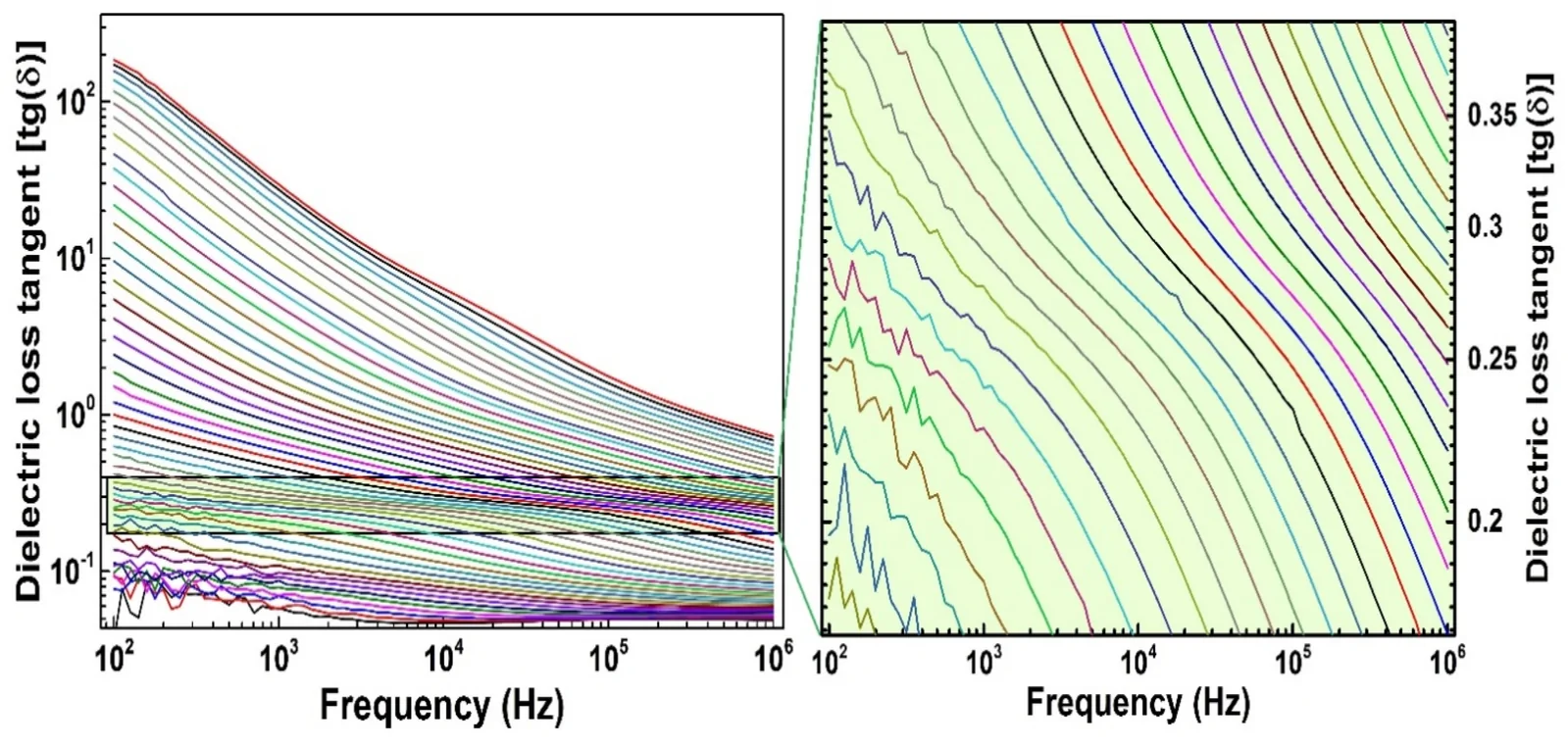
Frequency dependence of the dielectric loss tangent (tg(delta)) at different temperatures of the Bi_0.8_Ba_0.2_FeO_3_ compound.

The relaxations peaks are expected to appear clearly in the dielectric loss tangent (tg(δ)) vs. frequency curves at different temperatures. Figure 6 illustrates the representative curves of the dielectric loss tangent as a function of frequency and temperature. Pronounced relaxation peaks are observed to shift to higher frequencies proportionally to temperature, validating the presence of thermally activated relaxation processes. Importantly, it was observed that the dielectric loss tangent values corresponding to the relaxation process of the studied compound are extremely low (≤0.5). Accordingly, the very high dielectric constant and these low dielectric loss values confirm that the Bi_0.8_Ba_0.2_FeO_3_ compound is highly suitable for applications in capacitors and dynamic random-access memory [48,49,50].

#### 3.3.2. Imaginary Part of the Complex Modulus

**Figure 7:**
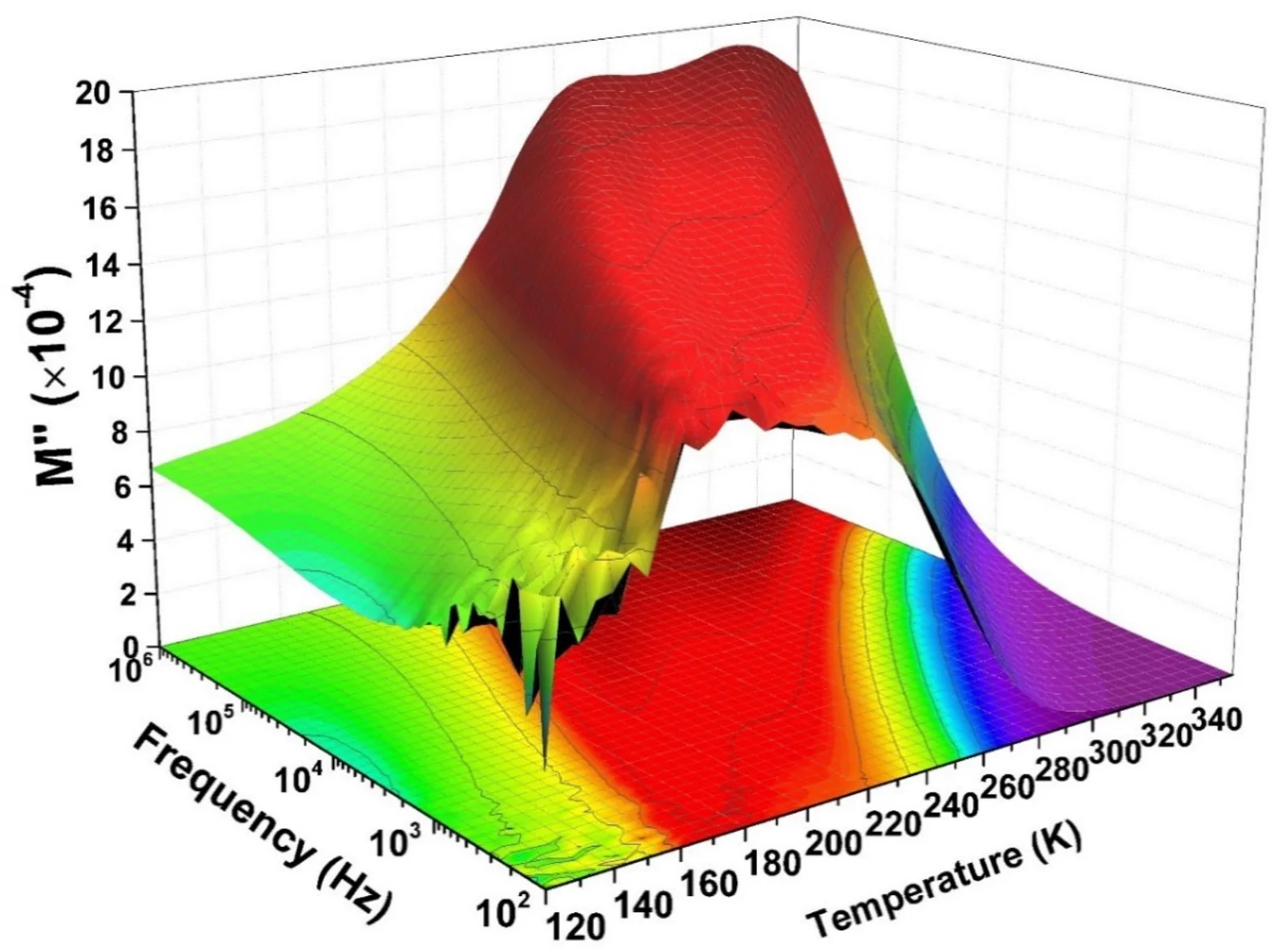
3D plot of the imaginary part M″ of the complex modulus (M*) as function of temperature and frequency of the Bi_0.8_Ba_0.2_FeO_3_ compound.

Two relaxation peaks for each temperature of the Bi_0.8_Ba_0.2_FeO_3_ compound are represented in the 3D plots of the imaginary part of the M*-complex curves (Figure 7). The first relaxation occurs in the low-frequency and low-temperature (LTDR) range, while the second one appears at higher frequencies (HTDR). The relaxation frequency associated with each peak increases with temperature, which aligns well with the thermally activated behavior of the relaxation process previously reported in perovskite ferrite materials [51,52,53].

The LTDR relaxation corresponds to the long-range hopping of charge carriers between neighboring sites across the capacitance of the grain boundary contribution characterized by high resistance, whereas the relaxation of the short-range hopping of charge carriers of highly conductive grains contribution appears at high frequency range (HTDR) [54]. For a deeper investigation of the relaxation process at low and high frequencies and to specify the adequate model of relaxation, we performed the adjustment of the imaginary part of the modulus in accordance with the Havriliak–Negami formalism (Equation (2)) [55]:

(2) $$M_{H\text{-}N}^{*}=\frac{1}{\varepsilon_{H\text{-}N}^{*}}=\frac{1}{\varepsilon_{\infty }+\frac{\varepsilon_{s}-\varepsilon_{\infty }}{{\left[1+{\left(i\omega \tau_{HN}\right)}^{\alpha }\right]}^{\beta }}}$$

The width and the asymmetry of M” curves are obtained from the alpha and beta shape factors, respectively, and the relaxation time is represented by τ_HN_. It was reported that the Cole−Davidson model (*CDRM*) is characterized by alpha equal to unit (α=1) and beta β≠1 [56]. When α = β = 1, the Debye model is the adequate for the relaxation process (*DRM*) [57]. Furthermore, when β = 1 and α≠1, the dielectric relaxation obeys the Cole−Cole model (*CCRM*) [58].

**Figure 8:**
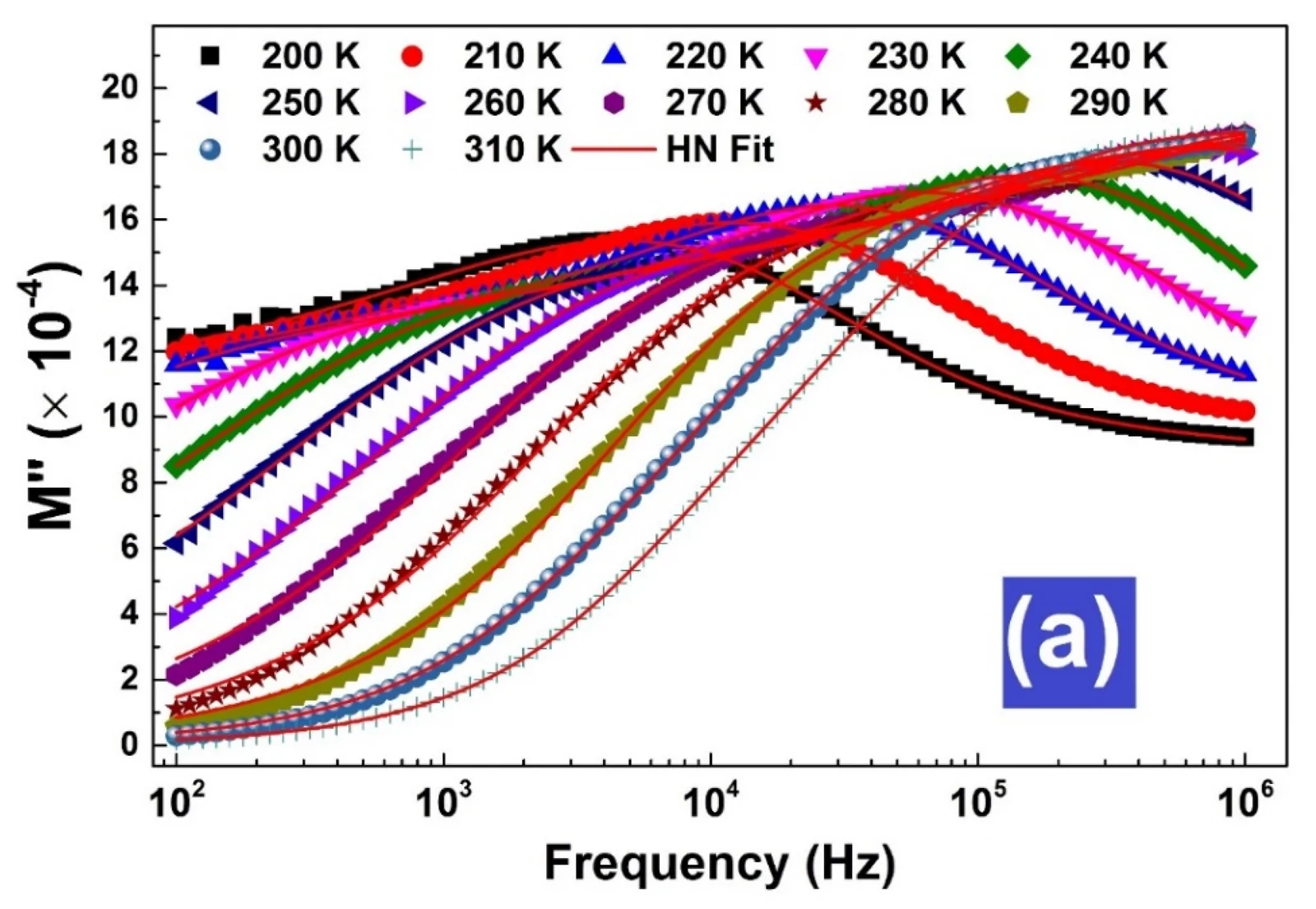

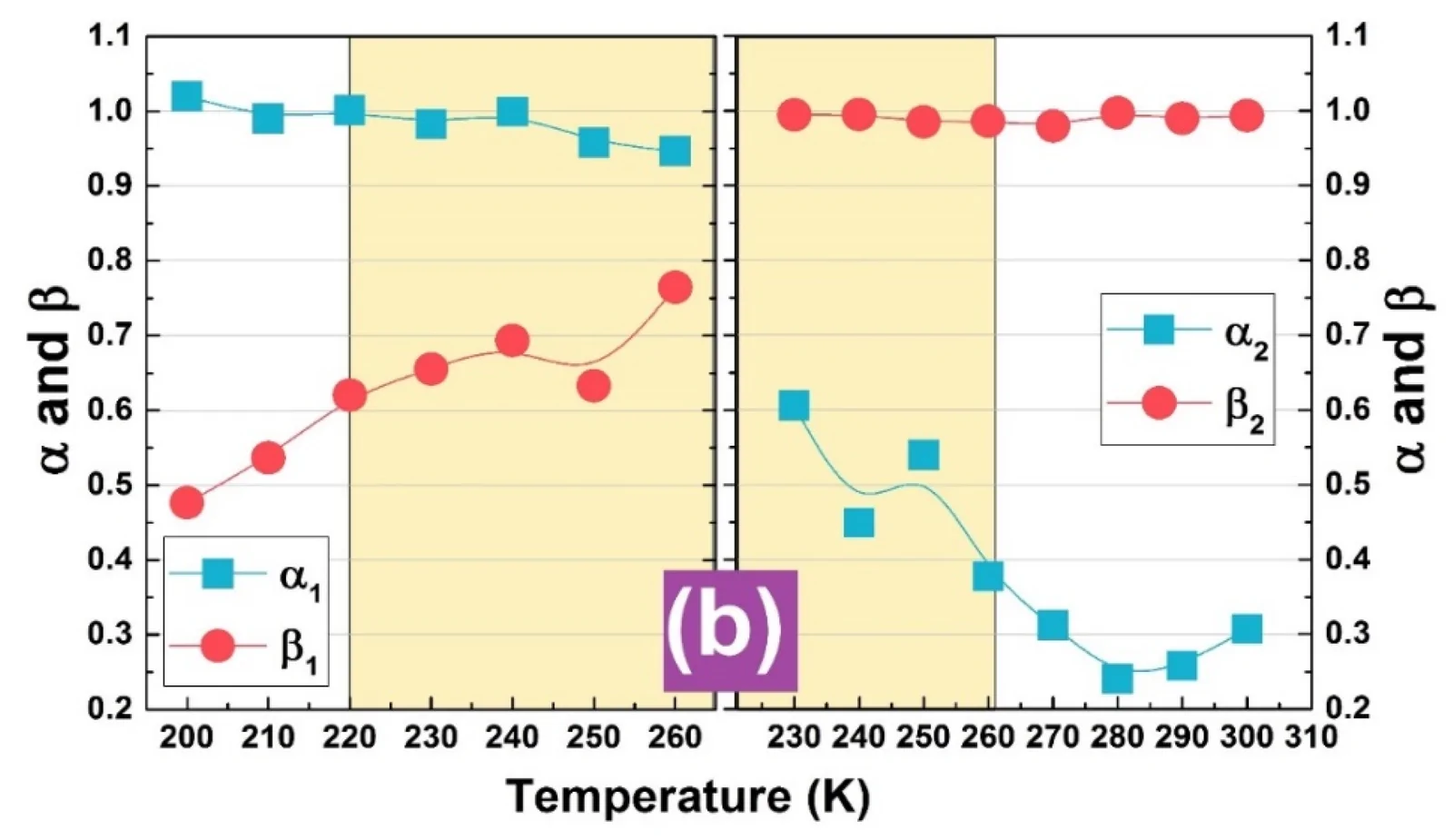
(**a**) Havriliak–Negami adjusted M” curves; (**b**) temperature dependence of the α and β parameters associated with low and high temperature relaxations of the Bi_0.8_Ba_0.2_FeO_3_ compound.

The refined Havriliak–-Negami parameters were obtained through numerical fitting of the imaginary part of modulus M” curves using MATLAB software (v. 24.1). The obtained fitted curves are presented in Figure 8a.

Upon examination of the temperature dependence of the values obtained for α and β (Figure 8b), we confirm that the CDRM characterizes the low temperature (LTDR) relaxation, which is attributed to grain contribution. Meanwhile, the relaxation at high temperature HTDR can be associated with the Cole–Cole model (CCRM) and related to the disordered groups across the grain boundary and the degree of their disorder [59].

### 3.4. Conductivity Study

**Figure 9:**
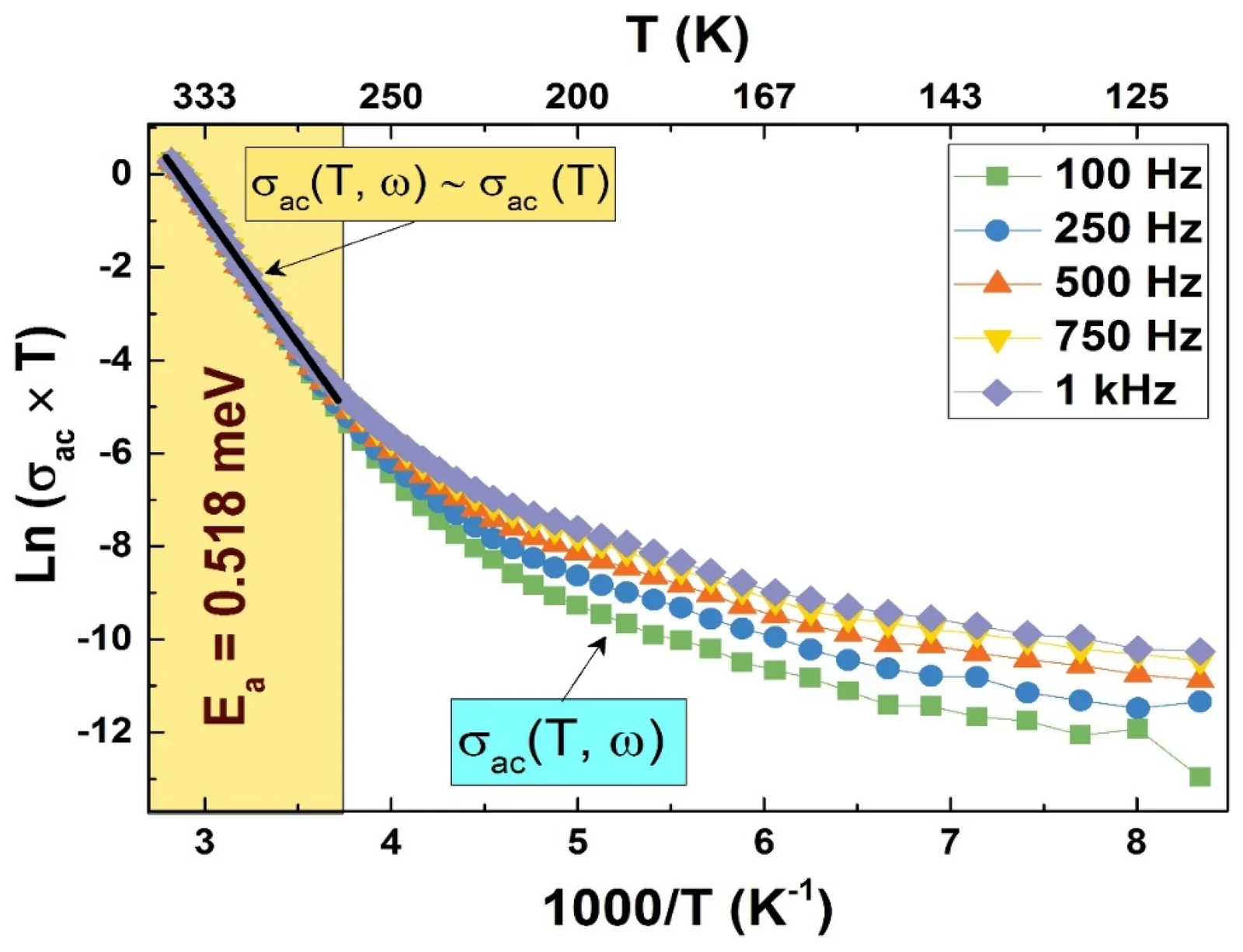
Ln($\sigma_{ac}\times T$) as a function of the inversed temperature for different frequencies of the Bi_0.8_Ba_0.2_FeO_3_ compound.

The AC conductivity behavior of Bi_0.8_Ba_0.2_FeO_3_ nanoparticles was studied through the logarithmic variation in the Ln (σ_ac_ × T) as a function of 1000/T, recorded at certain frequencies, as featured in Figure 9. The plot clearly reveals the presence of two distinct conduction regions characterized by different charge transport mechanisms.

At high temperature, the conductivity curves corresponding to various frequencies merge, suggesting that σ_ac_ becomes temperature-dependent but frequency-independent. This behavior confirms a thermally activated conduction mechanism by exhibiting an Arrhenius-type process [26,60]. The linear fit in this region yields an activation energy of approximately 0.518 eV, which is compatible with charge transport governed by the thermally induced migration of charge carriers linked to oxygen vacancies, which are commonly present in BiFeO_3_-based perovskite oxides [61,62,63]. Previous studies have also reported that this activation energy corresponds to the energy required for oxygen vacancies to move from one site to another [64,65].

In contrast, the LTR (low-temperature region) exhibits a strong dependence of alternative conductivity on both temperature and frequency, indicating a dispersive transport regime dominated by localized charge carriers. To further elucidate the conduction mechanism in this region, the experimental conductivity data were analyzed using the following equation [43]:

(3) $$\sigma_{ac}\left(\omega \right)=\left[\frac{\sigma_{s}}{1+\omega^{2}\tau^{2}}+\frac{\sigma_{\infty }\omega^{2}\tau^{2}}{1+\omega^{2}\tau^{2}}\right]+A\omega^{n}$$

**Figure 10:**
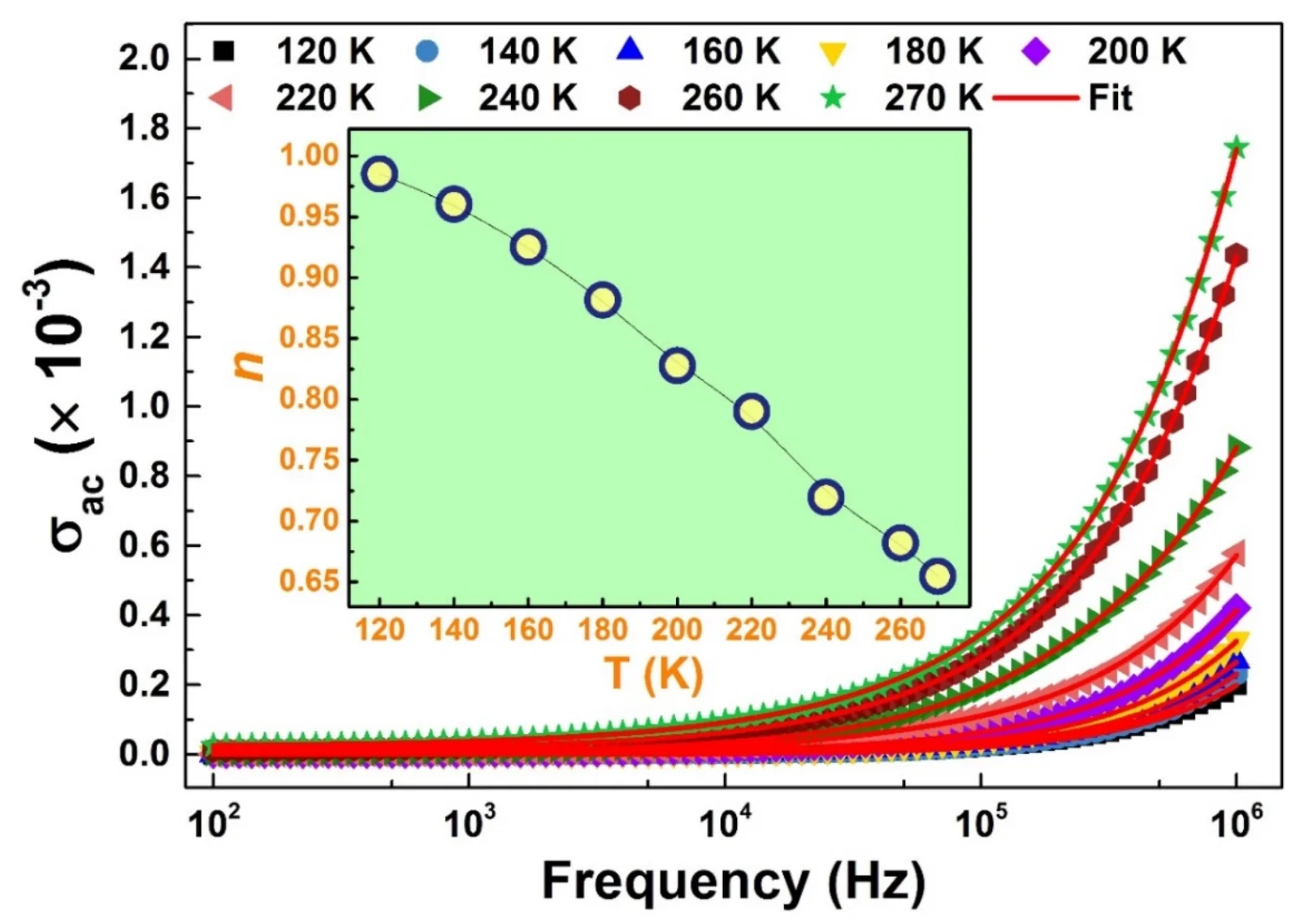
Fitted curves of the ac-conductivity at different temperatures of the Bi_0.8_Ba_0.2_FeO_3_ compound. The inset shows the power low exponent ‘n’ variation as function of temperature.

The first part, shown in brackets, represents the contribution of free charge carriers according to the Debye–Drude model [66]. It describes how the conductivity decreases at low frequencies (term $\frac{\sigma_{s}}{1+\omega^{2}\tau^{2}}$) and increases at high frequencies (term $\frac{\sigma_{\infty }\omega^{2}\tau^{2}}{1+\omega^{2}\tau^{2}}$), where $\sigma_{s}$ is the static conductivity, $\sigma_{\infty }$ is the high-frequency conductivity, *τ* is the relaxation time, and ω is the angular frequency. The second term, $A\omega^{n}$, is commonly referred to as the Universal Dielectric Response (UDR). It accounts for the contribution of localized or trapped charge carriers, where A is a constant and n is an exponent typically ranging between 0 and 1. The parameter n provides important insight into the nature of charge transport, indicating that the conduction process arises from the hopping of charge carriers between localized states. Its temperature dependence reveal models such as correlated barrier hopping (CBH), overlapping large polaron tunneling (OLPT), non-overlapping small polaron tunneling (NSPT) or quantum mechanical tunneling (QMT) models. These models have been extensively used to describe Ac conduction in doped multiferroic and ferrite materials [67,68,69,70,71]. A fitting analysis was carried out on the ac conductivity data of the studied compound. The experimental and fitted curves are shown in Figure 10, with the temperature dependence of the n parameter shown in the inset. The observed systematic decrease in the parameter n with increasing temperature supports the applicability of the correlated barrier hopping (CBH) model as the dominant conduction mechanism.

## 4. Conclusions

In our study, Bi_0.8_Ba_0.2_FeO_3_ nanoparticles were prepared via the sol–gel route. The structure and crystallization of the prepared compounds were evaluated by XRD measurements, showing a P/4mmm tetragonal structure with a secondary phase corresponding to the Pbam space group. Magnetic measurements confirmed the enhanced remanent magnetization and saturation magnetization of the prepared material compared with bulk BiFeO_3_. Nevertheless, despite this improvement, the relatively modest absolute saturation magnetization does not support its classification as a high-performance permanent-magnet material. Therefore, the magnetic properties of Bi_0.8_Ba_0.2_FeO_3_ should be considered primarily in the context of multifunctional and magnetoelectric applications. The dielectric constant, dielectric loss, modulus and the AC conductivity data were carried out over a frequency and temperature range of 10^2^ to 10^6^ Hz. The enhanced dielectric response, combined with the reduced dielectric losses and improved electrical stability induced by Ba^2+^ substitution, highlights the potential of Bi_0.8_Ba_0.2_FeO_3_ nanoparticles as a promising candidate for future dielectric-based electronic applications, including memory-related devices such as DRAMs. The modulus analysis confirms the presence of relaxation phenomena and the contributions of grains and grain boundaries in the transport properties. The AC conductivity analysis proves that the CBH model is the dominant conduction mechanism for our sample. Based on these findings, Bi_0.8_Ba_0.2_FeO_3_ nanoparticles can be considered promising candidates for multifunctional capacitors, magnetic sensors, and magnetoelectric devices, owing to their enhanced dielectric and multiferroic properties.

## Figures and Tables

**Table 1:** XRD profile refinement results of the Bi_0.8_Ba_0.2_FeO_3_ compound.

Phase	%	Space Group	a (Å)	b (Å)	c (Å)	V (Å^3^)
Bi_0.8_Ba_0.2_FeO_3_	97.78	*P4/mmm*	3.973_6_	3.973_6_	4.028_1_	63.615
BaFe_2_O_4_ (impurity)	1.83	*Pbam*	17.426_3_	9.306_5_	10.870_7_	1763.024
Fe_2_O_3_ (impurity)	0.39	*Pbma*	5.684_8_	11.212_3_	7.817_1_	498.169

## Data Availability

The original contributions presented in this study are included in the article. Further inquiries can be directed to the corresponding authors.
